# Health outcomes of the Bolsa Família program among Brazilian Amazonian children

**DOI:** 10.11606/s1518-8787.2020054001519

**Published:** 2020-01-23

**Authors:** Katherine J Ford, Barbara Hatzlhoffer Lourenço, Fernanda Cobayashi, Marly Augusto Cardoso

**Affiliations:** I École des Hautes Études en Santé PubliqueRennesFranceÉcole des Hautes Études en Santé Publique (EHESP), Rennes, France; II Universidade de São PauloEscola de Saúde PúblicaDepartamento de NutriçãoSão PauloSPBrasilUniversidade de São Paulo. Escola de Saúde Pública. Departamento de Nutrição. São Paulo, SP, Brasil

**Keywords:** Bolsa Família, Human Capital, Childhood Growth, Brazilian Amazon, Child Development, Poverty, Income, Social Programs, Government Programs, Social Capital

## Abstract

**OBJECTIVE:**

One of the primary objectives of Brazil’s conditional cash transfer program, *Bolsa Família*, is to break the intergenerational transmission of poverty by improving human capital via conditionalities. In this study, we hypothesized that health indicators of *Bolsa Família* participants would be comparable to those of other local children who were nonparticipants after two years of follow-up in the city of Acrelândia, Acre state, Western Brazilian Amazon.

**METHODS:**

Data from a population-based longitudinal study were analyzed to examine school enrollment, vaccination coverage, height and body mass index for age z-scores, and biomarkers of micronutrient deficiencies (iron and vitamin A) between *Bolsa Família* participants (n = 325) and nonparticipants (n = 738).

**RESULTS:**

Out of 1063 children 10 years and younger included in the 2007 baseline survey, 805 had anthropometric measurements and 402 had biochemical indicators in the 2009 follow-up survey. Prevalence rate ratio (PRR) for non-enrollment in school at 4 years of age was 0.58 (95%CI: 0.34–1.02) when comparing *Bolsa Família* participants with nonparticipants. No difference was found for vaccination coverage, which was insufficient for most vaccine-preventable diseases. *Bolsa Família* participants were less likely to show a positive change in body mass index for age z-scores compared with nonparticipants (PRR = 0.81, 95%CI: 0.70–0.95), while a positive change in height for age z-scores was similar in the groups. No differences in micronutrient deficiencies were found between groups after 2 years.

**CONCLUSIONS:**

Early school enrollment and consistent nutritional indicators between *Bolsa Família* participants and nonparticipants suggest *Bolsa Família* was facilitating similarities between groups over time.

## INTRODUCTION

Poverty is a pervasive condition that limits the development of human capital, which encompasses the skills, knowledge, and health needed by members of a society for potential economic progress^1^. In the context of children’s health, achieving ideal linear growth and weight gain in the first two years of life have meaningful connections with educational attainment^2^ and thus with human capital development. Inadequate nutrition during childhood can impact physical growth, cognitive development, susceptibility to infections, and school performance, perpetuating cycles of inequity in the long term^3,4^.

Brazil has seen dramatic changes in economic growth and poverty pervasiveness. In 1983, Brazil’s poverty prevalence, defined as less than R$100/person/month (~34USD/person/month^a^), sat at 38% of the population. Between 1983 and 2006, Brazil halved this prevalence to 19%^5^. Accelerated reductions after 2001 are owed in part to important redistributive policies implemented by the Brazilian government, including *Bolsa Família *(BF), a conditional cash transfer program^5^. The BF program was commissioned in 2003 with two primary objectives: to address the acute needs of poor households, and to break the intergenerational transmission of poverty by improving human capital via education and health conditions^6^.

In 2006, families earning less than R$120/month (~55USD/month^b^) with children younger than 17 years received regular monthly payments by the BF program. The payments were conditional on children aged 0–6 years receiving recommended vaccinations and regular growth monitoring, and children aged 6–15 years maintaining an 85% school attendance rate. The extreme poor received an additional unconditional payment — those earning less than R$60/family/month (~28USD/family/month^b^) — regardless of the presence of children in the household^6,7^. Monthly payments ranged between R$15 and R$95 (~7–44USD^b^) depending on the severity of poverty and family composition^7^.

The BF program has shown important impacts on health indicators in children from poorer segments of society. Research has identified decreases in under-five mortality rates — particularly from malnutrition and diarrhea — reductions in under-five hospital admission rates, better vaccination coverage for children under 1 year old, more consistent growth monitoring, and better psychosocial health in older children (7-17 years old)^8^. However, scientific literature on *Bolsa Família* and child nutritional status, including biomarkers of dietary intakes, on an individual level versus a household level is sparse^11^. Furthermore, BF is managed in a decentralized manner at the municipal level^12^, thus assessing associations between BF payments and outcomes of interest in children from poorer families throughout different municipalities is important. As far as we know, studies on the nutritional status of Brazilian children and BF participation in an Amazonian context are limited to one, which was specific to a rural population and used multiple indicators of economic transition to identify changes in anthropometric measures over time^13^.

In this study, we analyzed school enrollment and vaccination coverage, as well as prospectively assessed nutritional status indicators in children from Acrelândia, an Amazonian municipality, according to enrollment in the BF program. We hypothesized that nutritional status indicators would be comparable between BF participants (BFP) and nonparticipants (NP), likely facilitated by improved dietary intakes with the regularized payments. These hypotheses considered that Acrelândia is relatively impoverished as a municipality compared with Acre state and Brazil as a whole^14^, thus comparisons between BFP and NP were posited to be comparisons between the poor and those marginally better-off economically.

## METHODS

### Study population and data collection

This is a population-based longitudinal study of children in Acrelândia, Acre, with baseline data collection in 2007. The municipality is located in the western part of the Amazon forest. Acrelândia had 12,538 inhabitants in 2010^14^. Roughly 64% of the population was vulnerable to poverty in 2010, though this had decreased from 74% in 2000. The percentage of children classified as extremely poor also dropped from 35% to 24% over the same time period^14^. Despite some improvements in these indicators of vulnerability, the Acrelândia human development index (HDI = 0.604) continued to be lower than that of the country (HDI = 0.727)^14^.

In December 2007, all families with children 10 years and younger, residing in the urban area of Acrelândia were invited to participate in the study. Field procedures were previously described^15,16^. Firstly, a structured, pilot-tested questionnaire was completed with face-to-face interviews with the child’s parents or guardians by trained members of the research team. The questionnaire included questions about socioeconomic characteristics, housing conditions, parental characteristics, schooling, child’s health care utilization and morbidities. A short food frequency questionnaire (FFQ) was used for estimating food group consumption within the last month (fruit, green vegetables, root vegetables, dairy, beans, meat, eggs and fish) with parents of children aged 24 months and older. Answers were divided into categories: monthly or less (≤ 3 times per month), weekly (1–6 times per week), and daily (≥ 1 time per day). Children younger than 24 months were excluded from this analysis because the protocols for measuring their food intake were different and this early period is a transition time for habitual food intake when infants are weaned from breast milk on to the range of foods typical of the family’s diet^17^.

BF program participation was self-reported and confirmed by presentation of a BF registration card. Vaccinations were verified in medical records. Afterwards, parents were invited to take their child for a physical examination at the local health clinic, where anthropometric measurements and fasting venous blood samples were taken. For this analysis, subjects were excluded if information on BF participation was missing or if a blood sample was not taken for the 2007 baseline survey.

In December 2009, a follow-up survey was conducted with anthropometric assessment among all participants and blood collection in a subsample of children older than 6 years. The 2007 procedures and equipment were repeated in 2009.

Trained research assistants took height and weight measurements using standardized equipment and measurement protocols^18^. Two height measurements were taken and recorded to the nearest millimeter using stadiometers (model 208; Seca, Hamburg, Germany). Weight was measured in duplicate and recorded to the nearest 100 g using an electronic scale (model HS-302; Tanita, Tokyo, Japan). Mean values were used for calculating height and body mass index (BMI), which were subsequently transformed into height for age z-scores (HAZ) and BMI for age z-scores (BAZ) using WHO AnthroPlus software^19,20^.

Hemoglobin concentrations were measured onsite at a field laboratory using an automated cell counter (ABX Micro 60; Horiba, Montpellier, France). The blood samples were then frozen at -70°C on dry ice and shipped to São Paulo, where they were subsequently analyzed within 6 months of collection. Plasma ferritin and soluble transferrin receptor concentrations were assessed using commercially available enzyme immunoassays (Ramco, Houston, Texas). Anemia was defined as hemoglobin concentration < 110 g/L for children younger than 60 months and < 115 g/L for those 60 months or older^21^. Iron deficiency was defined as plasma ferritin levels < 12 µg/L for those younger than 60 months and < 15 µg/L for those 60 months or older, or transferrin receptor concentrations > 8.3 mg/L (Ramco Laboratories, 2014)^22^. Children who were both anemic and iron deficient were classified as having iron deficiency anemia^16^. Retinol concentrations were measured using standard high-performance liquid chromatography methods (Shimadzu, Kyoto, Japan). Vitamin A deficiency was defined as serum retinol concentrations ≤ 0.70 µmol/L^23^. All laboratory analyses assayed internal and external blinded quality control specimens in each run, with inter-assay coefficients of variation within 7%.

### Statistical analysis

Summary characteristics were assessed using χ^2^ tests or Fisher exact tests for categorical variables, while *t*-tests were used with continuous variables with normal distributions. A wealth index was created using principal component analysis based on 12 household items, as a proxy for economic status over the long term^24^. The relationship of BF with conditionalities was explored using Poisson regression models with robust variance. School non-enrollment was assessed from 4 and 6 years of age. Vaccination coverage was explored in children 24 months and older because most of the childhood vaccinations recommended by the Brazilian Ministry of Health are administered before the age of 15 months^25^. Vaccination coverage for a vaccine-preventable illness (VPI) was considered complete if the recommended number of doses was administered before 24 months — adequate timing of the doses was disregarded. Poisson regression models were fitted for coverage for all of the 8 VPIs, as well as for ≥ 6 VPIs.

Covariates of interest were added to each model in hierarchical levels of child characteristics, socioeconomic and housing conditions, and maternal characteristics. For each level, a threshold of p < 0.10 was used to keep variables in the model. In all models, the wealth index was maintained regardless of p-value, since BF is a program to alleviate poverty by income transfers. Many of the eligible poor are not in the program while some who are ineligible are receiving it^6^, thus controlling for variations in wealth is important.

Children with information on BF participation at baseline and anthropometric assessments completed in 2007 and 2009 were included in the prospective anthropometric analysis, with comparison of mean HAZ and BAZ in 2007 and 2009 by groups, and between the BFP and NP at both time points. Positive growth in HAZ and BAZ, defined as a positive change in z-scores from 2007 to 2009, was modeled using Poisson regression models with the above described methods. Stunting, defined as HAZ < -2^18^, was compared with χ^2 ^tests. Children with information on BF participation in 2007 and blood samples in both years were included in the prospective biomarker analysis. Prevalence of nutritional deficiencies were compared within groups in 2007 and 2009, and between groups at both time points. Noting that BFP and NP groups in 2009 are based on BF status in 2007 is also important as BF status precedes changes over the two years. All statistical analyses were performed using Stata version 11.0 (StataCorp, College Station, TX, USA).

The study protocol agrees with the guidelines laid down by the Declaration of Helsinki for research involving humans. Informed consent was collected from all parents or guardians before enrollment and data collection. The ethics review board of the School of Public Health at the *Universidade de São Paulo* approved all procedures involving human subjects.

## RESULTS

Among the 1250 children eligible to participate in 2007, 1225 (98%) were enrolled in the study. Parents or guardians of 1151 children (92% of those eligible) completed the questionnaire. Another 88 children were excluded due to missing BF information (n = 30) or a missing blood sample (n = 58). The sample in our 2007 study included 1063 children, 31% of which were receiving BF. There was no difference in the mean age (p = 0.884), skin color proportions (p = 0.478), sex (p = 0.310) and mean wealth index (p = 0.961) between those excluded from baseline and those included. Out of those excluded for not having a blood sample in 2007, no differences in BF participation were observed compared with those who had blood samples in 2007 and were included (p = 0.453). The 2009 follow-up assessment included 805 children for anthropometric analysis, 76% of the initial sample. The 402 children included in the 2009 biomarker analysis represented 38% of the initial sample, as only those over 6 years old were eligible for the 2009 blood collection.

Table 1 describes general differences between the two groups at baseline. Mean wealth index among BFP was significantly lower than among NP. BFP were older and lived in more crowded houses; their mothers were older, and their parents had less schooling time. They were smaller in z-scores for height and BMI and were more likely to have had malaria in the past year. Some differences in the skin color of the children were observed, though the population was predominantly brown. Among the children with white skin (n = 95), 15.8% were BFP, compared with 37.5% of children with black skin (n = 48) and 32.1% of those with brown skin (n = 853).

**Table 1:** Characteristics of the study children according to Bolsa Familia program participation in 2007, Acrelândia, Western Brazilian Amazon.

Variable	Non-participants*			Bolsa Familia participants*			p-value^†^
	
	n	Mean or %	95% CI	n	Mean or %	95% CI	
*Child characteristics:*							
Female sex	738	49.7		325	50.8		0.755
Skin color	689			307			0.003
black		4.4			5.9		
brown		84.0			89.3		
white		11.6			4.9		
Age (in years)	738	4.6	4.4–4.8	325	6.4	6.1–6.7	0.000^‡^
<5 years		57.1			30.8		0.000
5-10 years		43.0			69.2		
*Socio-economic conditions:*							
Wealth index	737	0.12	-0.16–0.40	325	-1.04	-1.43–0.66	0.000^‡^
Wealth index (*median*)		1.27			-0.67		
Number of persons/bedroom	737	2.5	2.4–2.6	325	3.1	2.9–3.2	0.000^‡^
Untreated water	713	8.4		312	6.7		0.358
Non-enclosed sewage system	730	82.6		315	89.2		0.007
*Parental characteristics:*							
Mother’s age (in years)	738	29.0	28.4–29.7	325	32.8	32.0–33.7	0.000^‡^
Mother’s years of schooling	715	7.1	6.8–7.3	313	4.7	4.3–5.1	0.000^‡^
Father’s years of schooling	569	6.8	6.5–7.1	236	4.8	4.3–5.2	0.000^‡^
*Baseline health parameters*:							
BMI for age z-scores	729	0.05	-0.02–0.12	324	-0.20	-0.31–0.09	0.002^‡^
Height for age z-scores	730	-0.29	-0.37–0.21	324	-0.47	-0.58–0.37	0.010^‡^
Diarrhea in past 15 days	736	25.0		323	21.7		0.243
Malaria in past 12 months	566	0.9		228	3.1		0.022

CI, confidence interval. BMI, body mass index.

* Totals may differ from the total number of study children due to missing values.

† Pearson x^2^ test.

‡ *t*-test.

Among children 24 months and older, more than 75% of participants in both groups consumed meat, milk and beans on a daily basis, while less than 25% consumed vegetables and leafy greens on a daily basis, and less than a third had fruits on a daily basis. The only significant difference in frequency of intake between the two groups were that BFP reportedly consumed more beans on a daily basis (88% vs 81% for NP; p = 0.008; data not shown in tables).

Among children 4 years and older, 12.1% of NP were not enrolled compared with 6.1% of BFP (p = 0.015). With adjustment for child’s age, skin color, wealth index, and mother’s years of schooling, prevalence of non-enrollment in school from 4 years of age among BFP was 42% higher than NP (PRR = 0.58, 95%CI: 0.34–1.02). This is significant at a p < 0.10 level. When considering children 6 years and older, 6.5% of NP were not enrolled compared with 2.7% of BFP (p = 0.105). From 6 years of age, the prevalence rate of non-enrollment in school among BFP children was similar from that of NP (PRR=0.46, 95%CI: 0.18–1.18). Results were adjusted for child’s age in years and wealth index (data not shown in tables).

None of the children had complete coverage against all of the eight VPI. There was also no difference in immunization coverage for each of the eight VPI between groups. Overall, for both groups, tuberculosis and yellow fever had the best coverage at 97.9% and 95.1%, respectively. Rotavirus had the worst coverage at 12.1% for NP and 11.9% for BFP. After adjustment for child’s skin color, wealth index, having an enclosed sewer system, mother’s age and mother’s years of schooling, no difference was found between BFP and NP (PRR = 0.94, 95%CI: 0.78–1.14) for missing coverage against more than two VPI (data not shown in tables).

In analyzing growth over follow-up, although mean values of HAZ were significantly higher in NP in comparison with BFP in both assessments, no difference was found for prevalence of positive changes in HAZ from 2007 to 2009 scores between those receiving BF at baseline and those who were not (Table 2). Proportions of stunted children were also similar between the two groups at both points (4.3% of NP vs 4.4% of BFP in 2007, p = 0.961; 3.6% of NP vs. 6.4% of BFP in 2009, p = 0.076; data not shown in tables). On average, NP were heavier than their counterparts in both assessments. The prevalence ratio of positive changes in BAZ was 0.81 (95%CI: 0.70–0.95, Table 2) for BFP in relation to NP, meaning they were less likely to have positive changes over the two years. These findings were adjusted for baseline age in years, baseline BAZ, having an enclosed sewage system, and baseline wealth index.

**Table 2:** BMI for age z-scores and height for age z-scores between 2007 and 2009 by *Bolsa Família* participation in 2007, Acrelândia, Western Brazilian Amazon.

		2007		2009		Positive change in z-score over two years
		
		NP	BFP	NP	BFP	PRR
Height for age z-score (*n *= 805)	Mean (95%CI)	-0.23 (-0.32–0.14)	-0.44 (-0.56–0.32)	-0.36 (-0.44–0.28)	-0.55 (-0.66–0.43)	1.07 ^†^ (0.87–1.31)
BMI for age z-score (*n *= 804)	Mean (95%CI)	0.13 (0.04–0.21)	-0.23 (-0.36–0.10)	0.24 (0.15-0.34)	-0.17 (-0.30–0.03)	0.81 ^††^ (0.70–0.95)

BMI, body mass index. CI, confidence interval. NP, nonparticipant. BFP, *Bolsa Família* participant. PRR, prevalence rate ratio.

^† ^Poisson regression adjusted on baseline HAZ, baseline age in years, and wealth index.

^†† ^Poisson regression adjusted on baseline BAZ, baseline age in years, enclosed sewage system, and wealth index.

Table 3 shows the prevalence of micronutrient deficiencies based on biomarker concentrations by group. In 2007, BFP had a lower prevalence of iron deficiency than NP. Over the two-year period, the prevalences of iron deficiency, iron deficient anemia, anemia, and vitamin A deficiency were improved in both the NP and BFP groups. In 2009, nutritional indicators were similar between NP and BFP.

**Table 3:** Nutritional deficiencies of study children in 2007 and 2009 by *Bolsa Família* participation in 2007, Acrelândia, Western Brazilian Amazon.

	NP at baseline (n = 246)		p-value^*^	BFP at baseline (n = 158)		p-value^†^	NP & BFP in 2007 p-value^‡^	NP & BFP in 2009 p-value**
	
	2007 N (%)	2009 N (%)		2007 N (%)	2009 N (%)			
Anemia	18 (7.4)	0 (0.0)	0.000^††^	14 (8.8)	0 (0.0)	0.000^††^	0.634	N/A
Iron deficiency	87 (36.1)	13 (6.7)	0.000	42 (26.4)	8 (5.6)	0.000	0.043	0.675
Iron deficiency anemia	10 (4.2)	0 (0.0)	0.001^††^	6 (3.8)	0 (0.0)	0.015^††^	0.851	N/A
Vitamin A deficiency	30 (13.0)	11 (5.0)	0.003	19 (12.3)	5 (3.4)	0.005^††^	0.839	0.605^††^

NP, nonparticipant. BFP, *Bolsa Família* participant. N/A, non applicable.

* Pearson X^2^ test between NP in 2007 and 2009.

^†^ Pearson X^2^ test between BFP in 2007 and 2009.

^‡^ Pearson X^2^ test between NP and BFP in 2007.

^**^ Pearson X^2^ test between NP and BFP in 2009.

^†† ^Fisher’s exact test.

## DISCUSSION

To our knowledge, this is the first population-based study on the evidence of BF outcomes in peri-urban Amazonian children. Our study looked at anthropometric changes between BFP and NP over time, rather than looking at BFP before and after they are enrolled in the program as previously done with the rural Amazonian population^13^. Furthermore, this study offers additional insights into the nutritional status of children receiving BF by using biomarker information and FFQ surveys to give a thorough picture in a particular context.

In this study, the baseline characteristics of BFP and NP suggest BF payments were going to the poorer and less educated families in our population — those with less potential for social mobility. In 2007, the Brazilian criteria for accessing BF was a monthly income of less than R$120 (~62USD^c^)^6^. Among the NP in our study, 8% were drinking untreated water, 83% were living in a house without proper sewage infrastructure and some of their parents still had few years of schooling. Despite BF payments going to the poorer and less educated families on average, many NP were also living in poor housing conditions and had mothers with fewer years of schooling.

In terms of education, children from poorer families in Brazil are more likely to be enrolled in school later, repeat grades and dropout from school early^7^. The BF program is said to improve school attendance and reduce dropout rates, though there is little ground for conclusions on improved school performance^6,7^. Our study among children from 4 years of age suggests BF may work in terms of enrolling children in school early, although no variables examined educational progression nor the quality of education, which tends to be lower in areas highly served by conditional cash transfer programs^26^.

BF had no association with vaccination coverage in our study, even though vaccination coverage is a condition for receiving the benefit. This corroborates previous research on a national scale describing no differences in vaccination coverage between BFP and NP^27^. A potential explanation for the low but similar vaccination rates of BFP and NP could be an insufficiency of primary care services. The North Region of Brazil, where Acrelândia is located, was noted to have had slower expansion of the primary health care changes made by the government in the mid-1990s^28^.

Positive changes in HAZ during follow-up were similar in the groups. However, BFP had fewer positive changes in BAZ. In the context of Acrelândia, anthropometric differences remained between groups and BF was probably not sufficient to affect growth outcomes during a 2-year follow-up period. The importance of growth, particularly in the first two years of life, should be considered when interpreting these results, given they provide a critical window for optimizing growth patterns and minimizing health risks and human capital in the long term^2^.

Biomarkers of nutritional status at the 2-year point showed no significant difference in this study. Noticeably, both groups (BFP and NP) improved in the same micronutrient deficiencies, suggesting that both groups had similar access to foods contributing to their vitamin A and iron levels. Besides the finding related to similar food group intakes between BFP and NP at baseline, BFP families had enough resources to procure comparable diets in terms of micronutrients to that of NP over time. Given that an estimated 87% of BF families report that their funds go primarily towards food^12^, our results support the notion that food procurement in BFP is likely similar to their wealthier NP counterparts in Acrelândia.

Some limitations of this study should be considered. Firstly, our study failed to capture improvements in literacy and numeracy skills with schooling — that which is beneficial for the development of human capital. Our study suggests BF is working in terms of enrolling children in school early, although no variables examined educational progression or more importantly the education quality, which is particularly important in poorer regions^26^. Secondly, our observations could possibly reflect BF parents being more accustomed to education and health outcomes of interest to the program due to the conditionalities, which may in turn induce some response bias, particularly for school enrollment, as this was self-reported. As no randomization would be possible in such context, more studies are important to confirm our findings. Thirdly, we did not explore BF outcomes in relation to the value of the transfers given. BF is a binary variable in our study; therefore, nuances with the total amount given and levels of deprivation are not thoroughly explored.

In summary, the two groups were similar between themselves regarding anemia, iron and vitamin A deficiencies and positive changes in HAZ scores over the 2-year follow-up period. These results agreed with our hypothesis that nutritional status indicators would be prospectively comparable between BFP and NP. The prevalence of positive changes in BAZ were less marked with BFP. Some similarities pointed to BF as a successful program for developing human capital in poor children to the level of their better-off peers, but additional investigation is worthwhile.
